# Student perspectives on climate change and sustainability education in England: experiences and expectations

**DOI:** 10.14324/111.444/ucloe.3341

**Published:** 2025-07-10

**Authors:** Nicola Walshe, Joy Perry, Grace Healy

**Affiliations:** 1 Centre for Climate Change and Sustainability Education, IOE - Faculty of Education and Society, UCL, UK

**Keywords:** education, climate change, sustainability, children and young people, the environment, outdoor learning, student autonomy, green careers, health and wellbeing, educational inequalities

## Abstract

This paper presents findings from a large-scale survey of 2429 students in Years 7–9 (ages 11–14) in England, exploring their experiences and expectations of climate change and sustainability education. The study reveals that while most students learn about climate change and sustainability in school, primarily through geography and science lessons, there is a desire for a more comprehensive and engaging approach. Students express a strong interest in learning about the future impacts of climate change, practical solutions and global perspectives. They emphasise the importance of outdoor learning, hands-on activities and opportunities for meaningful participation in school and community initiatives. The findings highlight the need for climate change and sustainability education to be embedded across the school curriculum, to provide opportunities for outdoor learning, and to nurture student agency. Explored within a framework of children’s rights to education and wellbeing and respective inequalities, this research provides valuable insights for stakeholders seeking to improve climate change and sustainability education and empower young people to address the climate and environmental crisis.

## Introduction

This paper is a response to two prevailing points at issue: first, high-quality climate change and sustainability education (hereafter referred to as CCSE) is widely agreed as being imperative for equipping children and young people with the knowledge, skills and capabilities needed to navigate the myriad of existing and predicted climate and environmental challenges [1]; second, the education sector must respond swiftly to develop the engaged global citizens on whom our planet’s livelihood depends [2]. Given their foundational role in shaping societal values and future trajectories, schools hold a clear responsibility to embed CCSE as a core element of their educational mandate. While children and young people feel much of the responsibility for ‘solving’ the climate and environmental crisis, little research has explored school students’ understandings and expectations of CCSE; yet, developing high-calibre CCSE reasonably should consider how it is experienced by learners. In response, this paper presents selected results from the UCL Centre for Climate Change and Sustainability Education (CCCSE)’s large-scale survey of school students in England, with a specific focus on Years 7–9 (ages 11–14) – a period during which students engage with a broad range of compulsory subjects as part of the Key Stage 3 National Curriculum [3]. To begin, we explore the curriculum context of CCSE within England, before outlining its placement within a framework of school responsibility and going on to consider student perspectives.

### Climate change and sustainability education and its curriculum context in England

The notion of ‘climate change education’ (CCE) extends beyond a basic understanding of scientific processes and the causes and impacts of climate change [4], to encompass a more holistic and action-oriented approach that develops learners’ capacity to critically engage with – and respond to – the climate crisis. Contemporary definitions (e.g., [2,5,6]) emphasise that CCE should not only include the transmission of knowledge, but also foster emotional engagement, ethical reflection and the development of agency to help mitigate climate-related issues. It is increasingly understood as a multidisciplinary and transformative discipline that builds students’ competencies to participate in personal, societal and political responses to climate change. ‘Sustainability education’, sometimes referred to as ‘education for sustainable development (ESD)’ (e.g., The Education and Teaching Foundation [7,8]), historically focuses on the interconnectedness of social, economic and environmental systems, and how to live in a way which ‘meets the needs of the present without compromising the ability of the future generations to meet their own needs’ [9]. While the teaching and learning of climate change and sustainability can vary in scope and emphasis, both involve critical systems thinking, problem-solving skills and active modes of citizenship that are necessary for creating a more sustainable future. In practice, effective CCSE integrates these elements, offering a comprehensive and interdisciplinary framework for understanding the challenges and opportunities facing our planet.

This study specifically focuses on the experiences and expectations of students aged 11–14 in England who are regulated by the Department for Education (DfE).^1^ The National Curriculum in England features climate change explicitly only in secondary science, with no mention of sustainability at all. In 2022, the DfE implemented a Sustainability and Climate Change Strategy [11] which outlines priorities for England across five action areas (Climate Education, Green Skills and Careers, Education Estate and Digital Infrastructures, Operations and Supply Chains, and International) and three initiatives (National Education Nature Park, Climate Action Awards and Sustainability Leadership). While laying important groundwork for schools to behave more sustainably, enhance teacher practice and provide suitable career guidance for students, the strategy leaves major areas open to provision and individual responses by school leaders and perhaps, as a result, has been termed a ‘placebo policy’ [12]. Further, the DfE’s current conceptualisation of CCSE and related policy is quite narrowly focused on the teaching and learning of science ‘to ensure all young people receive high-quality teaching on the scientific facts about climate change and environmental degradation’ ([11], n.p.). This is problematic, as it undermines the notion that ‘all disciplines contain knowledge and skills that can contribute towards developing the complex, interdisciplinary understandings of the environmental crisis and how we can live more sustainably’ ([13], n.p.). Further, UCL CCCSE’s national survey of teachers [14] illustrates how little teachers are formally supported in their professional development of CCSE and the consequences this has on student experiences in schools. As such, the educational landscape in England is such that it leaves significant numbers of young people at risk of leaving its system without learning necessary climate- and sustainability-related knowledge, skills and capabilities.

### Climate change and sustainability education and its placement within a framework of school responsibility

The extent to which CCSE can inform English curriculum rests in a large part on how it is framed within the education sector’s obligations to ensure equity, inclusion and wellbeing. This entails moving beyond the technical transmission and measurement of knowledge and understanding and speaks to the broader purposes of schooling for the betterment of the community. Education systems possess remarkable leverage against the climate and environmental crisis and must be appropriately utilised as instruments in climate change mitigation and adaptation, capable of driving transformative societal change [13,15]. Through CCSE, schools can exercise what Cavanagh [16] describes their moral responsibility to help shape what society and its institutions most value: in instituting a *culture of care*, schools fulfil their function as forums for societal critique, exciting new ideas and continually enquiring ‘what is appropriate for these children in these circumstances?’ (p. 20).

One of the core features of CCSE is addressing the inequalities caused or exacerbated by the climate and environmental crisis. Educational interventions must consult, for instance, the uneven distribution of climate-related knowledge across different educational levels observed in UK households [17]. This is because higher education levels are associated with increased climate policy support and enhancing one’s capability to lead a low-carbon lifestyle [17]. Individuals from more advantaged socio-economic backgrounds will often experience greater educational success, an advantage which usually accumulates over time, while income inequalities are compounded with disparities among gender, ethnicity and disability [18–20]. From a health perspective, professionals are beginning to understand the physical and mental tolls of climate change on children and young people’s wellbeing, and are looking to education for ways of transforming, for instance, climate-related anxiety into climate-related agency [21]. Professional bodies for addressing this demographic’s wellbeing have provided explicit warnings for stakeholders, outlining the growing rates of depression and anxiety in what is an extraordinary epidemic for younger generations [22,23]. As of 2023, research on behalf of the NHS reported that 1 in 5 children and young people aged 8–25 years old had a probable mental disorder, while over half (54.8%) of young people aged 17–25 years experienced climate change-related distress [24]. It is an unfortunate reality that climate change and sustainable development are now a part of a broader conversations around health and inequality, and its ramifications for students’ wellbeing must be considered in conjunction with other risk factors (e.g., social media and poverty levels). Evaluated in this way, CCSE is not only a subject to be learnt, but also a mechanism through which schools can uphold a richer framework of societal responsibility and education.

### Student perspectives and improved schooling for climate change and sustainability education

There is a scarcity of research, within and beyond the English context, which considers student perspectives on CCSE and subsequent inequalities. Polls have indicated that students retrieve much of their information in this area from school, social media, television programmes and their families; however, they suggest that students do not feel adequately informed in ways which lead to meaningful sustainability, and desire more practical learning opportunities which enables them to act [25]. Students report learning about climate change predominantly in geography lessons, although they feel that these activities insufficiently relate to contemporary and local examples of social, economic and political impact [26]. Important findings in England have shown that, while students generally seek to learn more about climate change- and sustainability-related topics (e.g., resource depletion and pollution), girls and those from more advantaged households convey stronger interest than boys and those from less advantaged households [27]. However, despite increasing recognition of the need for learner-informed educational strategies, empirical studies capturing students’ direct insights into CCSE remain limited. Identifying gaps such as these are key to developing high-quality and equitable CCSE; this research aims to begin to achieve this.

The literature emphasises multiple advantages to including students’ views in education, not only for the improvement of educational practices but as a matter of upholding children’s right to voice themselves freely in matters which affect them, as per the United Nations Convention on the Rights of the Child [28]. When students participate in decision-making processes about their learning, they exhibit greater degrees of ownership in their education [29], provide valuable insights which inform teaching and learning practices [30] and positively impact their school environment and its academic achievement [31]. Although research as it pertains exclusively to CCSE is limited, findings such as those from Torsdottir et al. [32] provide frameworks for incorporating students as active participants in school sustainability-related efforts, ones which are strengthened by an explicit connection to children’s rights. This perspective is in keeping with Riley’s suggestion that students can behave as ‘active agents of change’ rather than passive recipients of their education ([33], p. 125); their experiences and creative energies can help generate productive and enduring change in schools (p. 109) – change which the current education system needs in fulfilment of CCSE.

How a sufficiently engaged student body behaves as part of CCSE in England remains to be entirely understood; opportunities for dynamic staff–student collaboration are evident, for example, in Dunlop et al.’s [34] process of inclusive and collective manifesto-making for setting educational priorities for environmental sustainability. When paired with recent surveys of teacher perspectives in England (e.g., [14]), gathering student views has the potential to help establish some consensus in achieving principles of CCSE and respective teaching and learning alignment. This study aims to supplement this understanding of the student role by focusing exclusively on their attitudes, preferences and ambitions regarding the education of climate change and sustainability in schools [3]. The two research questions that frame this research paper are: *what are student experiences and perceptions of CCSE in school?* and *what are student expectations for CCSE?*

## Materials and methods

This section describes the methods of data collection and analysis, as well as ethical considerations and limitations of the research.

### Data collection

Students were surveyed to gather their experiences of and perspectives on climate change and sustainability through an online questionnaire administered via the Qualtrics platform. The survey was developed through a collaborative, iterative process involving drafting, team discussions and testing, followed by final refinements based on a pilot study conducted with one class of Year 8 students (n = 32). The questionnaire design and content were informed by existing research and national surveys (e.g., [35–38]) to ensure relevance and rigour. A variety of question types were employed – including multiple-choice, Likert scales measuring agreement or disagreement, frequency scales and open-text fields – to facilitate participant engagement and maximise insight.

The questionnaire comprised seven sections:

1. All about you
2. What do you think about climate change and sustainability?
3. What do you do about climate change and sustainability in school?
4. What do you think about climate change and sustainability in school?
5. What would you like to learn about in the future?
6. How do you feel about nature?
7. All about your life

The initial section, ‘All about you’ comprised a series of demographic questions pertaining to school year group, gender and ethnicity, as well as three questions aiming to elicit information with regards to students’ level of advantage (how many books or eBooks at home, access to the Internet at home, and whether parents/carers went to university). This allowed us to explore potential differences between groups of students as part of our analysis.

### Student recruitment

Responses were sought from students in England in Years 7–9 (ages 11–14), between March and May 2024. These years were chosen because they represent a critical development stage during which young people form increasingly sophisticated worldviews and begin to engage more independently with societal issues. Moreover, this age range falls within Key Stage 3, a phase in which students study a compulsory National Curriculum [39], making it particularly suitable for examining cross-curriculum integration of CCSE. Participants were recruited via their teachers who were, in turn, contacted via a range of networks, social media channels and existing communication distribution lists. Efforts were made to gather responses in schools with varied experiences of CCSE; although it is possible that teachers who are already engaged in teaching related to climate change and sustainability may have supported the survey, the research sought to encompass whole classes or year groups such that responses were received from students with a wide variety of perspectives and engagement.

### Participants

Full details of participants are presented in Walshe et al. [3]; in summary, responses were received from 2429 students from 30 schools across England, including state-funded (24) and independent (6) schools. The sample of students comprised 40% in Year 7, 34% in Year 8 and 27% in Year 9. Of those students who responded, 56% were girls, 43% were boys and 1% were non-binary and gender diverse students. In relation to ethnicity, the sample of students comprised 56% with White backgrounds; 25% with Asian/Asian British backgrounds; 7% with mixed backgrounds; 5% with Black/Black British backgrounds; 4% with Arab backgrounds; and 4% with other ethnicities/backgrounds.

The questionnaire followed international surveys, such as the Trends in International Mathematics and Science Study [40] in asking students to report numbers of books or eBooks at home. In England, students’ reports of numbers of books at home has been found to inversely scale with their eligibility for free school meals; students with the most books at home have the lowest proportion of students eligible for free school meals, while students with the fewest books at home have the highest proportion of students eligible for free school meals [41]. Considering differences between those with very few books and those with the most books offers insight into the extent of potential educational and wider inequalities. Our sample of students involved 16% with the fewest books at home [‘None or very few (0–10 books)’] and 25% with the most books at home [‘Enough to fill three or more bookcases (more than 200)’].

### Data analysis

We applied a range of approaches to quantify students’ responses and to reveal similarities or differences between students with the most and fewest books. For quantitative data, this comprised cross-tabulations with chi-squared tests and independent-samples t-tests (without assuming equal variances across the groups of students being considered), where equivalent results emerged across different analytical approaches and perspectives. For qualitative data, students were asked to finish the sentence: *I would improve the teaching of climate change and/or sustainability at secondary school by …* with free-text comments. The process of thematic analysis of this data comprised data familiarisation, inductive coding of the data by one author, discussing, defining and naming themes across the author group, then writing the findings. The inductive coding was completed manually, allowing themes to be applied to the data as it was reviewed, rather than interpreting using a pre-defined framework. This was an iterative process which involved returning to the original data and the literature several times (Braun et al. [42]). This grounded, constructivist approach allowed for the emergence of themes which were then considered in the light of the literature [43]. Examples of how data were applied to specific themes are given in Table 1.

**Table 1.** Indicative quotes assigned to the three key themes emerging from the student free-text responses to: *I would improve the teaching of climate change and/or sustainability at secondary school by* …

Theme	Indicative quotes
Exploring climate change and sustainability across a wider range of subjects	Having art or design lessons where we aren’t taught about the effects of climate change, and instead reuse old plastics and things that would ordinarily be thrown away to make art pieces from, in order to be more sustainable. Making it part of the whole curriculum and properly informing and educating teachers about the subject so that the stuff we learn is true and helpful to us.
Physical engagement with nature and the outdoors	I would improve the teaching of climate change in secondary schools by learning it physically. By going outside and studying it like that makes it enjoyable, and easier to understand. Having more times outside with nature during the school day as we barely ever go outside.
Opportunities for school-based and broader community action	Enforcing more action-related activities rather than just speaking to give students a sense of the power they have. Having more proactive projects, less learning about the background and focus on making a difference with change.

### Ethical considerations

The research was conducted with approval from the Institutional Research Ethics Committee prior to the commencement of data collection and voluntary, informed consent was obtained from all participants at the start of the questionnaire. Data was managed in accordance with the UK GDPR and DPA 2018. Participant data was anonymised before analysis. Questionnaires were administered predominantly by teachers during lessons; as such, it was an important part of the consent process to explicitly state that the decision to complete the questionnaire (or not) would have no bearing on the students’ progress or attainment in school.

### Limitations of the research design

While every effort was made to contact students across England, the reach of the survey was limited by our own networks; as such, most responses were received from students within London and the South-East. This means that the demographic composition of the sample may not fully reflect the diversity of the student population in terms of socio-economic status, ethnicity and school type, which may limit the generalisability of the findings. Further work is needed to ensure full geospatial representation across England, as well as other jurisdictions of the UK: Wales, Scotland and Northern Ireland.

The survey was intentionally focused on students aged 11–14, a group within Key Stage 3 and during which most students still study all National Curriculum subjects; further research exploring the perspectives of other age groups, including both primary aged children (4–11 years), those studying GCSEs (14–16 years) and A Level students (16–19 years), would allow a more nuanced understanding across the student body. Additionally, the questionnaire was administered at one point in time in spring 2024; while this is useful for presenting a snapshot, a longitudinal study would better understand developments in students’ understandings and perspectives, thereby allowing further analysis of factors which support engagement with climate change and sustainability.

Finally, the data are based on self-reported responses, which may be subject to personal or recall biases. This means that while students’ insights offer valuable perspectives, they may not always correspond precisely with classroom realities. This limitation is compounded with the possibility that comparisons made between students with the fewest books (16%) and those with the most books (25%) reflect the extremes of the total sample, thereby potentially skewing data interpretation.

## Results

This section firstly considers student articulations of their experiences and perceptions of CCSE in school (RQ1), before going on to explore what students told us they would like to learn about in CCSE in school in both quantitative and qualitative responses (RQ2).

### What are student experiences and perceptions of climate change and sustainability education in school?

**Figure 1:**
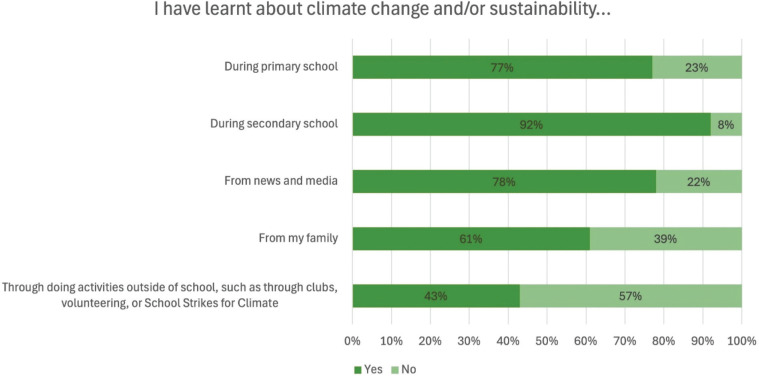
Student learning about climate change and/or sustainability. The figure shows the percentage of students who selected each option.

As shown in Fig. 1, 92% of students reported learning about climate change and/or sustainability during secondary school, 78% from news and media, 77% during primary school, 61% from family and 43% through doing activities outside of school. While this highlights the significant role of formal education in schools as providing students with equity in access to learning opportunities around CCSE, it also emphasises the ways in which students will have differing starting points and connections around this in-school learning based on whether they are also engaged in learning about climate change and sustainability in other spaces. Statistically significant differences exist between students with the fewest and most books; fewer students with the fewest books report learning about climate change and sustainability during primary school, from news and media, from family or in activities outside school (Table 2). This further emphasises the importance of school learning and raises questions about the barriers to learning about climate change and/or sustainability for some students.

**Table 2.** Student learning about climate change and/or sustainability compared between students with the fewest and most books

I have learnt about climate change and/or sustainability …	All students	Comparison			
		Fewest books	Most books	Cohen’s D	Sig. (*p*)
During primary school	77%	72%	80%	**0.187**	**0.011**
During secondary school	92%	88%	92%	0.126	0.090
From news and media	78%	67%	83%	**0.371**	**<0.001**
From my family	61%	36%	78%	**0.960**	**<0.001**
Through doing activities outside of school, such as through clubs, volunteering, or School Strikes for Climate	43%	30%	49%	**0.409**	**<0.001**

The table shows the percentage of students who selected ‘Yes’ (rather than ‘No’). The results are presented across all students within the sample (‘All students’), and across groups of those with different extents of books at home (where ‘Fewest books’ refers to ‘None or very few (0–10 books)’ and ‘Most books’ refers to ‘Enough to fill three or more bookcases (>200)’), and the magnitude (‘D’; Cohen’s D) and statistical significance [‘Sig. (*p*)’; *p*-value] of the difference across those with different extents of books at home. Significant *p*-values (*p* < 0.05) and the associated magnitudes are highlighted in bold and green shading for clarity.

**Figure 2:**
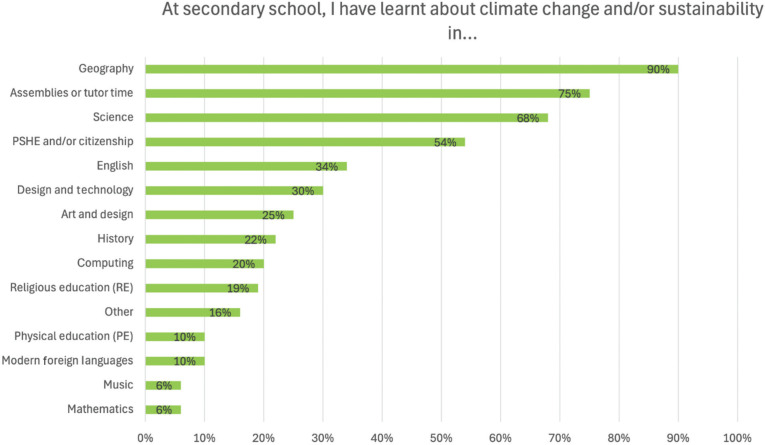
Secondary school subjects in which students have learnt about climate change and/or sustainability. The figure shows the percentage of students who selected each option.

Students reported learning about climate change and/or sustainability more often in geography (90%), assemblies or tutor time (75%), science (68%) and personal, social, and health education (PSHE) and/or citizenship (54%) (Fig. 2). Several subjects were far less likely to provide students with opportunities for learning on climate change and/or sustainability, especially music (6%), mathematics (6%), physical education (10%) and modern foreign languages (10%). Other subjects with lower rates of inclusion according to students were religious education (19%), computing (20%), history (22%) and art and design (25%). Students were also invited to specify ‘other’ subjects, with most responses referring to drama and food technology/food science. This pattern aligns with where climate change and sustainability are found within the National Curriculum in England [44,45] and where teachers report teaching of climate change and sustainability takes place in England [14]. Statistically significant differences exist between students with the fewest and most books for some subjects, but this includes students with fewest books reporting learning about climate change and/or sustainability more frequently in some subjects and less frequently in other subjects (Table 3).

**Table 3.** Secondary school subjects in which students have learnt about climate change and/or sustainability compared between students with the fewest and most books

At secondary school, I have learnt about climate change and/or sustainability in …	All students	Comparison			
		Fewest books	Most books	Cohen’s D	Sig. (*p*)
Assemblies or tutor time	75%	69%	75%	0.119	0.102
Art and design	25%	19%	26%	**0.156**	**0.030**
Design and technology	30%	24%	33%	**0.201**	**0.005**
English	34%	41%	26%	**0.320**	**<0.001**
Geography	90%	82%	90%	**0.229**	**0.002**
History	22%	26%	16%	**0.266**	**0.001**
Computing	20%	27%	17%	**0.260**	**0.001**
Mathematics	6%	6%	5%	0.060	0.422
Modern foreign languages (e.g., French or Spanish)	10%	14%	9%	0.149	0.051
Music	6%	6%	5%	0.062	0.415
Personal, social, and health education (PSHE) and/or citizenship	54%	46%	59%	**0.266**	**<0.001**
Physical education (PE)	10%	12%	7%	**0.194**	**0.013**
Religious education (RE)	19%	17%	20%	0.079	0.272
Science	68%	59%	73%	**0.293**	**<0.001**
Other (please add)	16%	18%	17%	0.026	0.796

The table shows response percentages and highlights significant differences (*p* < 0.05) and effect sizes (Cohen’s D) in both and green shading, consistent with Table 2.

**Figure 3:**
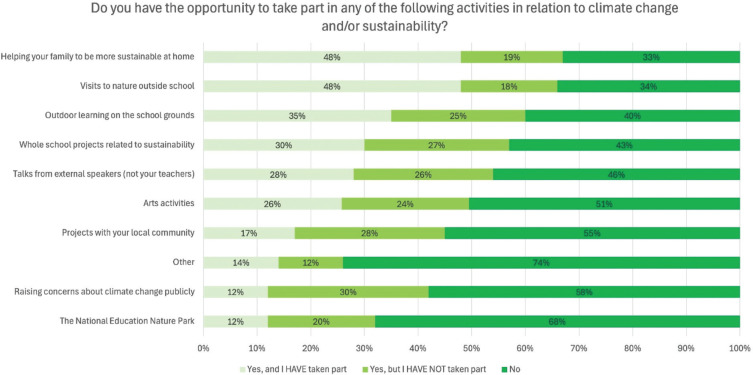
Student awareness of and engagement in a range of activities related to climate change and/or sustainability. The figure shows the percentage of students who selected each option.

Students were asked whether they had opportunities to participate in various CCSE activities. They could select whether the opportunity existed, and they had participated, the opportunity existed but they had not participated, or the opportunity did not exist. The most common activities students had the opportunity to engage in and had taken part in were ‘Helping your family to be more sustainable at home’ (48%) and ‘Visits to nature outside school’ (48%) (Fig. 3). Students reported limited opportunities to engage with the ‘National Education Nature Park’, ‘Raising concerns about climate change publicly’ or through undertaking ‘Projects with their local community’, with only 12%, 12% and 17% of students, respectively, participating in these activities. In ‘Other’ responses, students mainly referred to school-based learning (including through subjects, field trips, assemblies and tutor time), reading, researching, discussing climate change and sustainability and engaging with news or media on these topics. Students with most books were statistically more likely to engage in all activities than those with fewest books (Table 4).

**Table 4.** Student awareness of and engagement in a range of activities related to climate change and/or sustainability compared between students with the fewest and most books

		Yes, and I HAVE taken part	Yes, but I HAVE NOT taken part	No	Cramer’s V	Sig. (*p*)
Visits to nature outside school	Fewest books	34%	26%	40%	**0.214**	**<0.001**
	Most books	55%	14%	31%		
Helping your family to be more sustainable at home	Fewest books	29%	22%	49%	**0.330**	**<0.001**
	Most books	62%	14%	24%		
Outdoor learning on the school grounds	Fewest books	25%	30%	45%	**0.155**	**<0.001**
	Most books	40%	22%	39%		
Whole-school projects related to sustainability	Fewest books	20%	29%	51%	**0.172**	**<0.001**
	Most books	36%	26%	39%		
Talks from external speakers (not your teachers)	Fewest books	17%	31%	53%	**0.196**	**<0.001**
	Most books	34%	23%	43%		
Arts activities	Fewest books	16%	27%	56%	**0.152**	**<0.001**
	Most books	29%	20%	51%		
Projects with your local community	Fewest books	11%	27%	62%	**0.156**	**<0.001**
	Most books	22%	27%	50%		
Other (please add)	Fewest books	6%	14%	80%	**0.244**	**<0.001**
	Most books	22%	6%	72%		
The National Education Nature Park	Fewest books	10%	23%	68%	**0.100**	**0.017**
	Most books	12%	15%	73%		
Raising concerns about climate change publicly, e.g., public speaking, protesting or writing to an MP	Fewest books	8%	26%	66%	**0.109**	**0.008**
	Most books	15%	27%	59%		

The table shows response percentages and highlights significant differences (*p* < 0.05) and effect sizes (Cramer’s V) in bold and green shading.

**Figure 4:**
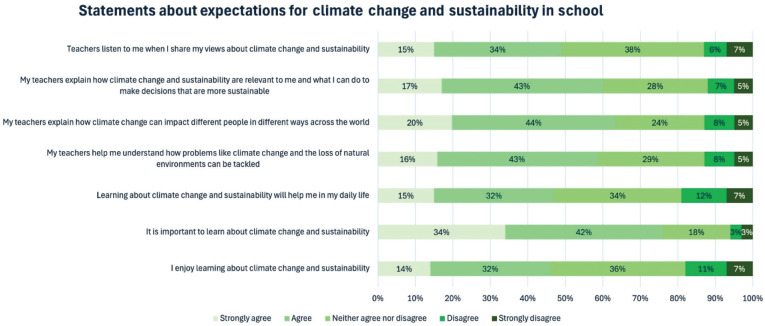
Extent of student agreement or disagreement with statements about climate change and sustainability. The figure shows the percentage of students who selected each option.

Students were asked to convey the extent of their agreement or disagreement with statements in relation to learning about climate change and sustainability more broadly. Students recognised the value of learning about climate change and sustainability, with 76% strongly agreeing or agreeing that it is important to learn about climate change and sustainability; however, only 46% strongly agreed or agreed that they enjoy learning about it and 47% strongly agreed or agreed that it will help them in their everyday life (Fig. 4). Sixty-four percent strongly agreed or agreed that their teachers explain how climate change can impact different people in different ways across the world. Sixty percent strongly agreed or agreed that their teachers explain how climate change and sustainability are relevant to them and what they can do to make decisions that are more sustainable. Fifty-nine percent strongly agreed or agreed that teachers help them understand how problems such as climate change and the loss of natural environments can be tackled. However, less than half (49%) of students strongly agreed or agreed that teachers listen to them when they share their views about climate change and sustainability. Students with the least amount of books at home are statistically more likely than those with the most books to show ambivalence around all statements about climate change and sustainability in school (Table 5).

**Table 5.** Student agreement with statements about climate change and sustainability, compared by number of books at home

		Strongly agree	Agree	Neither agree nor disagree	Disagree	Strongly disagree	Cramer’s V	Sig. (*p*)
I enjoy learning about climate change and sustainability	Fewest books	9%	21%	43%	12%	15%	**0.271**	**<0.001**
	Most books	19%	35%	31%	11%	4%		
It is important to learn about climate change and sustainability	Fewest books	19%	39%	31%	3%	7%	**0.329**	**<0.001**
	Most books	47%	36%	14%	1%	2%		
Learning about climate change and sustainability will help me in my daily life	Fewest books	10%	23%	41%	12%	14%	**0.234**	**<0.001**
	Most books	19%	34%	30%	12%	5%		
My teachers help me understand how problems like climate change and the loss of natural environments can be tackled	Fewest books	13%	33%	37%	7%	10%	**0.136**	**0.006**
	Most books	15%	42%	29%	9%	5%		
My teachers explain how climate change can impact different people in different ways across the world	Fewest books	13%	40%	31%	6%	9%	**0.173**	**<0.001**
	Most books	20%	44%	22%	10%	5%		
My teachers explain how climate change and sustainability are relevant to me and what I can do to make decisions that are more sustainable	Fewest books	14%	34%	37%	6%	10%	**0.176**	**<0.001**
	Most books	17%	45%	25%	8%	5%		
Teachers listen to me when I share my views about climate change and sustainability	Fewest books	12%	30%	41%	5%	12%	**0.126**	**0.015**
	Most books	19%	32%	37%	6%	7%		

The table shows response percentages and highlights significant differences (*p* < 0.05) and effect sizes (Cramer’s V) in both and green shading, consistent with Table 4.

### What are student expectations for climate change and sustainability education?

This section explores what students articulated should be included within CCSE within schools, drawing both on quantitative data and qualitative data from one open question within the questionnaire.

**Figure 5:**
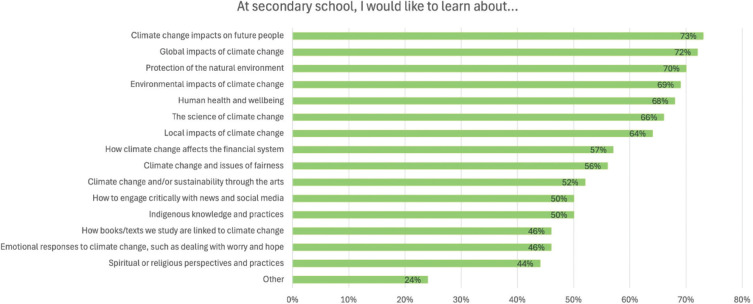
Climate change and sustainability themes that students would like to learn about. The figure shows the percentage of students who selected each option.

The questionnaire asked students to report whether they would like to learn about a range of topics in secondary school; responses are illustrated in Fig. 5. Topics that students were most likely to identify as being of interest were ‘Climate change impacts on future people’ (73%), ‘Global impacts of climate change’ (72%) and ‘Protection of the natural environment’ (70%). The least popular themes with students were ‘Spiritual or religious perspectives and practices’ (44%), ‘How books/texts we study are linked to climate change’ (46%), and ‘Emotional responses to climate change, such as dealing with worry and hope’ (46%). There were few responses within the ‘Other’ category; however, those students who responded commonly requested further information on potential impacts of climate change (e.g., ‘what will happen to the planet in the future’ and ‘how it affects people’) and on how they can make a difference (e.g., ‘how to help stop climate change’ and ‘how to influence others’). Statistically significant differences exist between students with the fewest and most books for all topics except ‘other’, with greater number of students with the most books reporting that they want to learn about all topics (Table 6).

**Table 6.** Climate change and sustainability themes that students would like to learn about compared by number of books at home

	All students	Comparison			
		Fewest books	Most books	Cohen’s D	Sig. (*p*)
Climate change impacts on future people	73%	55%	79%	**0.534**	**<0.001**
Global impacts of climate change	72%	55%	80%	**0.565**	**<0.001**
Protection of the natural environment	70%	51%	80%	**0.670**	**<0.001**
Environmental impacts of climate change	69%	52%	77%	**0.562**	**<0.001**
Human health and wellbeing	68%	58%	71%	**0.291**	**<0.001**
The science of climate change	66%	49%	73%	**0.509**	**<0.001**
Local impacts of climate change	64%	49%	71%	**0.466**	**<0.001**
How climate change affects the financial system	57%	44%	61%	**0.352**	**<0.001**
Climate change and issues of fairness	56%	39%	65%	**0.546**	**<0.001**
Climate change and/or sustainability through the arts	52%	39%	59%	**0.417**	**<0.001**
Indigenous knowledge and practices	50%	39%	55%	**0.329**	**<0.001**
How to engage critically with news and social media	50%	35%	59%	**0.500**	**<0.001**
Emotional responses to climate change, such as dealing with worry and hope	46%	33%	50%	**0.355**	**<0.001**
How books/texts we study are linked to climate change	46%	34%	55%	**0.419**	**<0.001**
Spiritual or religious perspectives and practices	44%	36%	46%	**0.205**	**0.006**
Other	24%	21%	28%	0.161	0.112

The table shows response percentages and highlights significant differences (*p* < 0.05) and effect sizes (Cohen’s D) in both and green shading, consistent with Table 2 and Table 3.

**Figure 6:**
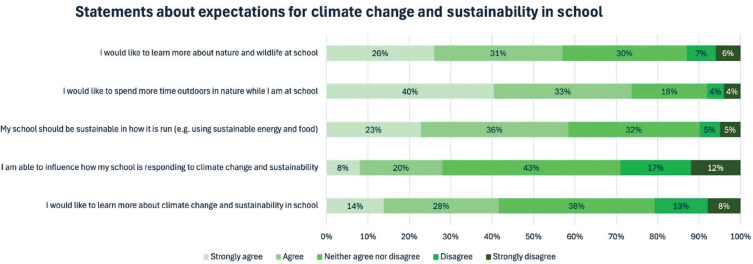
Percentage agreement with statements about student aspirations around climate change and sustainability education. The figure shows the percentage of students who selected each option.

Students were also asked the extent to which they agree with a range of statements pertaining to aspirations around climate change and sustainability (see Fig. 6). Forty-two percent of students agreed that they would like to learn more about climate change and sustainability in school. In relation to nature and the outdoors, 73% of students conveyed that they would like to spend more time outdoors in nature while at school and 56% of students said that they would like to learn more about nature and wildlife at school. Most students agreed that the school should be sustainable in how it is run (58%), whereas only 29% felt they were currently able to influence how their school is responding to climate change and sustainability. Table 7 shows there are statistically significant differences between the frequencies across the five response options between students with the fewest and the most books.

**Table 7.** Student aspirations around CCSE, based on agreement with various statements

		Strongly agree	Agree	Neither agree nor disagree	Disagree	Strongly disagree	Cramer’s V	Sig. (*p*)
I am able to influence how my school is responding to climate change and sustainability	Fewest books	7%	14%	49%	14%	17%	**0.151**	**0.001**
	Most books	8%	23%	39%	18%	12%		
I would like to learn more about climate change and sustainability in school	Fewest books	9%	18%	42%	14%	17%	**0.257**	**<0.001**
	Most books	21%	27%	36%	11%	5%		
I would like to learn more about nature and wildlife at school	Fewest books	15%	25%	40%	8%	12%	**0.277**	**<0.001**
	Most books	32%	35%	23%	5%	5%		
My school should be sustainable in how it is run (e.g., using sustainable energy and food)	Fewest books	13%	28%	43%	7%	9%	**0.337**	**<0.001**
	Most books	37%	35%	23%	2%	3%		
I would like to spend more time outdoors in nature while I am at school	Fewest books	28%	35%	24%	5%	8%	**0.218**	**<0.001**
	Most books	48%	30%	16%	3%	2%		

The table shows response percentages and highlights significant differences (*p* < 0.05) and effect sizes (Cramer’s V) in both and green shading, consistent with Table 4 and Table 5.

Through an open-text field, students were asked to finish the sentence*: I would improve the teaching of climate change and/or sustainability at secondary school by*…. Responses varied from short and nondescript to highly detailed. Some simply stated an imperative to teach it (e.g., ‘actually teaching it’ or ‘doing more’), or said they did not know (e.g., ‘I don’t know’ or ‘don’t know’); others elaborated further, allowing us to glean more nuanced information surrounding students’ views on CCSE. Responses were not analysed according to number of books at home, so cannot be used to draw socio-economic distinctions. Through the responses, students communicated a desire for increased opportunities to learn about climate change and/or sustainability through three themes: physical engagement with nature and the outdoors; exploring climate change and sustainability across a wider range of subjects; and opportunities for school-based and broader community action.

Firstly, a considerable number of responses centred on *exploring climate change and sustainability across a wider range of subjects*. Students’ written comments expressed concerns that the teaching of these issues is often confined to science and geography, while resting on tired or outdated resources, for example:

Making it part of the whole curriculum and properly informing and educating teachers about the subject so that the stuff we learn is true and helpful to us.

This could be in ways like designing ecologically and environmentally friendly structures in [design and technology] or seeing examples of self-sufficient ecosystems in person in biology – things like sealed terrariums.

Going past the scientific reasons of climate change, looking at the difference effects of climate change on a number of thing (e.g., finance, jobs, religious views).

Making it clear how it effects popular professional areas such as business and finance would motivate students to learn about it more and take it into consideration when going into particular professions. This is because more people now than ever are being drawn to entrepreneurship and related fields … it should be taught in schools in these areas to raise awareness [sic] of climate change in popular professions, that at first glance, may seem like they have nothing to do with the matter.

Students articulated the affordances for teaching climate change and sustainability across the curriculum for more project-based and action-oriented learning:

… incorporating a hands-on, interdisciplinary approach that combines science, social studies and ethics. This could involve project-based learning where students actively participate in local sustainability initiatives, use real-world data to analyse climate impacts and engage in discussions about policy and ethical considerations.

… not constantly focusing on the same, repetitive aspects of saving energy and reducing plastic waste, etc. We, as students, are already aware that plastic is bad. We know to turn the lights off when we leave a room. Don’t leave your appliances on standby, take shorter showers, recycle … this is basic knowledge – common facts. Constantly regurgitating this information doesn’t motivate young teenagers to become aware of or interested in the climate crisis … I believe that shifting the focus of eco-related lessons to the concept of innovation and encouraging pupils to think for themselves will be the most influential course of action.

Students also conveyed that the incorporation of climate change and sustainability across subjects further provides opportunities to incorporate global perspectives and approaches:

Talking more about the more nature-based, rural countries, and the ways their life is [impacted]. I wish to be able to learn more about the more spiritual and even religious theories [behind] climate change, and how different sectors of the Earth are affected by this. I would like to learn from people who rely on nature and Earth to survive, and their point of view.

Secondly, students articulated a demand for regular, timetabled *physical engagement with nature and the outdoors*, stressing the importance of outdoor enquiry for applying knowledge to appropriate physical contexts and for appreciating the varied beauty of wildlife, as well as for broader benefits for both learning and wellbeing. For example:

… help the children remember and have fun with [nature] rather than sitting in a classroom reading about it. Not all children concentrate in class properly and [may] forget most of the knowledge, but if you are outside, children will remember everything that was taught.

… have more outdoor lessons, e.g., science lessons are always indoors, even when we’re learning about leaves and ecosystems and nature – why not outside? It’s better for us, in so many ways!

… more outings and trips to areas related to nature so that we can explore new horizons, and to give these opportunities to everyone (or everyone who wants to take part) instead of a select few.

Finally, students articulated a desire for CCSE which incorporated *school-based and local action*. Their emphasis on applied learning demonstrated a recognition that the school community must lead by example, and that being afforded opportunities to participate in decision-making around their school or wider community environments would be both engaging and impactful. In practice, examples of student-led initiatives included:

A green team aiming to make the school zero carbon.

… allowing students to see how sustainable the school is, and how much work and effort it takes to be sustainable. (Is it easy to make a school sustainable or is it hard to make a school sustainable?)

Student suggestions include ordinary ‘green’ initiatives, including recycling, sourcing sustainable meals, a shared school allotment, reducing paper and electricity usage and exhibiting informational posters. But more than the enforcing of such eco-friendly policies, students highlight ways of more purposely embedding the issues of climate change into the mental fabric and philosophy of the community. In other words, introducing school-wide measures for provoking thought, opinion, and collective action:

Making it something we talk about more often, and when we discuss these things maybe we could have suggestions on how to make the school more sustainable, and those things could be taken into action.

Responses frequently included aspects of whole-school conversation and debate, informed by both students and the expertise of external speakers; special forums or assemblies, many suggest, would lend a refreshing dynamic to the topic, likely bringing about new ideas for implementing real change in the community. Participants speak about this not just in terms of generating new ways of learning and collaborating, but for the purposes of constructive policymaking and local government involvement.

… allowing us more creative output and more ways to share our ideas and views by giving us a more public point to do so. For example, school-wide debates (perhaps branching to other schools in the area) or allowing us to express our views through art and music pieces or composition.

## Discussion

Within this section, we consider three themes emerging from the data in relation to students’ experiences of, and expectations for CCSE. It should be noted that while our findings indicate significant socio-economic differences, further consideration is needed regarding how these align with existing research on socio-economic disadvantage within education and intersect with other significant educational inequalities (particularly ethnicity and gender). We recognise this as a limitation of the scope of this paper, and this will be explored in detail in further publications.

### Supporting climate change and sustainability education across the curriculum

A government-commissioned Curriculum and Assessment Review (CAR) in England was initiated in September 2024, aiming to consider whether the curriculum and assessment are ‘fit for purpose and meeting the needs of children and young people’ ([46], p. 1). As a response to years of campaigning to reform the existing 2014 version, CAR provides the opportunity to empower school communities to implement meaningful education around issues of climate change and sustainability. As such, this is a critical moment to re-conceptualise what CCSE should entail, its positioning within the realms of school responsibility and children’s rights, and the student perspective as a means through which a more nuanced and equitable understanding of this field can be achieved. Findings presented in this paper have implications for the structure and content of curriculum and assessment within England; compared with its counterparts, England’s current curriculum features a notably weaker framework for emphasising a broad range of skills across multiple and non-traditional subjects [47]. This currently lags behind its counterparts in Scotland, where *Learning for Sustainability* is an entitlement for all students and is embedded into the Professional Standards for Teachers [48], and Wales, where Education for Sustainability and Global Citizenship has been recognised as a priority since 2008 [49,50].

Examples from both England and internationally demonstrate that these challenges can be overcome. For instance, Rushton et al. [51] describe how case study schools in England were able to embed CCSE across the curriculum through whole-school planning and student-centred pedagogies. The research reported increases in student engagement, improved teacher confidence, and more equitable access to learning. Internationally, the Bicycle Model developed in Finland [6] has proven effective in targeting CCE into formal schooling through a balance of cognitive, emotional and action-oriented learning components. The model encourages cross-disciplinary approaches and has been adopted as a framework to support national education policy. Similarly, in Australia, Oliver et al. [52] highlights place-based and culturally responsive climate education programmes can successfully engage Indigenous students by integrating traditional ecological knowledge with Western scientific understanding. These examples demonstrate that CCSE can succeed even in rigid education systems when it is well-supported, co-designed and fully integrated into school life.

A persistent theme articulated by the students through our data is that high-quality CCSE should include interdisciplinary and innovative learning opportunities. Students reported learning about climate change and sustainability most prominently in geography (90%), assemblies or tutor time (75%) and science (68%); however, they conveyed interest in learning a broader range of themes, particularly regarding impacts on future populations and around the globe. Other areas of interest included effects of climate change on the financial system, justice-related matters, CCSE through the arts and Indigenous knowledge. While there are key concepts which children and young people need to understand, such as the interplay between greenhouse gas emissions and climate change [53], there is enormous potential for other subjects to complement and enhance the teaching and learning taking place in science and geography classrooms. As Power and Kitson argue, ‘Climate change is diagnostic, but climate change is not a “science” problem. It is a societal problem, a human problem. Technology-based “solutions” are not, as often depicted, cleanly separate from the messiness of real life, but will be developed and deployed according to (some) human values, priorities and judgements’ ([54], p. 2). These profoundly *human* features of CCSE necessitate multiple lenses through which to critically evaluate and respond to the crisis at hand. Against the backdrop of England’s traditionally subject-based education system, a revised curriculum must embrace a kind of organic fluidity, one which allows CCSE to move across all subjects and evolve according to new insight and cutting-edge developments.

Another significant finding from this study is that while 34% of students strongly agree that it is important to learn about climate change and sustainability (47% of advantaged students, as compared with 19% disadvantaged students), only 15% strongly agree that learning about climate change and sustainability will help them in their daily life (19% of advantaged students, as compared with 10% disadvantaged students). This not only illustrates socio-economic differences in the level of importance placed on CCSE but also reflects previous research which suggests that young people do not see the value of CCSE for their future, including for job opportunities. For example, findings of the broader survey found that few students think that CCSE will lead to increased work opportunity (31%), while even fewer express interest in pursuing climate change- or sustainability-related jobs (17%) [3]. Similarly, Hamlyn et al. [27] and Sheldrake and Reiss [55] reported that few students convey interest in jobs for mitigating climate change. This suggests that few students appreciate the importance and scope of future careers pertaining to climate change and sustainability.

These findings, therefore, suggest the need for a multidisciplinary approach in which CCSE is embedded across all school subjects. Such an approach would allow students to engage with multiple dimensions of the climate and environmental crisis and consider its immediate and long-term ramifications; this would give them opportunities for exploring a broader range of topics according to their interests and apply what they learn to various aspects of life, including future careers. CCSE has potential for revealing how green careers are applicable to multiple subjects (Gatsby Benchmarks; The Gatsby Charitable Foundation [56]), thereby enhancing green career pathways for students. Finally, CCSE across the curriculum would facilitate the use of socio-affective pedagogies for enabling transformative teaching and learning [12,57]. For example, affective approaches to CCSE, such as through art or narrative (e.g., Walshe and Tait [58]), can help students to cope with the complex emotions associated with these topics, while empowering them to act and advance efforts towards a more sustainable and equitable future [59]. This multidisciplinary approach would create more pathways for participation from students who are presently less engaged in CCSE (e.g., those of lower socio-economically circumstances) and, subsequently, contribute to mitigating educational inequalities.

### Developing student agency

This paper contributes to a theoretical framework of CCSE as a matter of children’s rights and achieving a more democratic schooling culture. These matters are inextricably related, as is supported by student views in this research and explicitly referenced as part of the UN’s authoritative guidance on delivering ‘transformative, inclusive, child-centred, child-friendly and empowering’ environment- and climate change-focused education [60]. While the UK Government ratified the UN Convention on the Rights of the Child in 1991, it has been criticised for failing to address and implement the observer committee’s nearly 200 recommendations for improvement [60] and for too-often excluding children’s views in its decision-making processes (Children’s Rights Alliance for England [61]). Although it is outside the scope of this paper to analyse the assorted reviews of education in England regarding its inequalities and performance (e.g., The Times Education Commission [62]), we take special notice of those pertaining to the empowerment of student voice and participation in school (e.g., Lundy et al. [63]) and subsequent implications for CCSE.

Our findings demonstrate that students are not only prepared to participate at multiple levels but expect increased involvement as part of achieving CCSE aims. Their qualitative responses especially shed light on ways in which they seek collaboration and their range of ideas for improving CCSE in their school communities. However, less than 30% of respondents agreed that they can influence how their school responds to climate change and sustainability, while less than half agreed that teachers listen when they express their views. Few students reported participating in activities which lend themselves to self-agency or action, such as projects with their local community (17%), those in collaboration with The National Education Nature Park (12%), or publicly rising concerns about climate change (12%). These findings are significant, as students themselves conveyed that participating in such activities contributes to their learning of CCSE and allows them to appreciate the potential for individual and collective action. And when students are encouraged to engage in meaningful ways, they are likely to respond more positively and constructively to the climate and environmental crisis [64,65].

Again, this study reveals noteworthy socio-economic inequalities; for each of the statistics listed above, even lower engagement in CCSE-related activity was gathered among students from lower socio-economic circumstances. This demographic was also less likely than their more advantaged peers to report enjoying learning about climate change and sustainability (30% and 54%, respectively) or perceive it as valuable (58% and 83%, respectively). These patterns may reflect broader issues of social inequality, where students from less advantaged backgrounds – often facing more immediate hardships, such as food or housing insecurity – are less likely to develop confidence and a sense of ownership around CCSE. These students may encounter systemic barriers within and beyond the school environment that constrain their participation, such as fewer school resources, limited adult support or minimal exposure to environmental issues outside the classroom. Interestingly, the qualitative dataset – comprising free-text responses of students’ ideas and perspectives – often presented more aspirational or agentic narratives than the quantitative data might suggest. This discrepancy likely reflects a skew toward more engaged responses from more advantaged students, who may feel more comfortable articulating their views and envisioning their role in climate action. The implication is that student agency – central to meaningful action in CCSE – is not evenly accessible to all. Instead, it is shaped by these intersecting socio-economic factors that affect students’ life experiences and opportunities. When schools fail to address such disparities, they risk reinforcing the very inequalities that education for sustainability seeks to challenge. This raises questions about school capabilities to facilitate student participation and enhance their attitudes about CCSE and their role within it. Because of existing constraints to student involvement beyond the formal curriculum [66], it is imperative to re-evaluate the scope and accessibility of these opportunities for all students (e.g., financial and transportation considerations) and improve measures for elevating marginalised voices.

With this in mind, we suggest the need for CCSE policy and practice to nurture student agency through opportunities for meaningful participation and action which consider both students’ perspectives and educational inequalities. The fulfilment of children’s right to a healthy and sustainable environment should include ‘access to information, participation in decision-making and child-friendly access to justice, with effective remedies, have equal importance to the empowerment of children, including through education, to become agents of their own destiny’ ([60], p. 12). And yet, this study suggests that students are not fully empowered to participate in CCSE, within or outside their schools, underscoring the need for actively involving children and young people to implement whole-school approaches to sustainability and collective decision-making (British Educational Research Association [67]; National Association for Environmental Education [68]). Further qualitative research would be beneficial to explore more thoroughly questions of *why* and *how* the teaching of climate change and sustainability are contingent upon demographic factors, refining gaps in our understanding and steering stakeholders towards increased evidence-informed policymaking.

### Nurturing nature connection

This study echoes prior studies illustrating how little outdoor learning activity takes place for young people, beyond physical education (e.g., Natural England [38]), an issue which is exacerbated by inequality. Despite its numerous benefits to students’ development, motor skills and socio-emotional behaviour [69], as well as engagement with standard subjects such as maths and science [70], opportunities for school-led outdoor learning have steadily decreased in recent years (e.g., Natural England [71]). While 73% of students in this study conveyed a desire for more outdoor learning in school, only 35% confirmed that they have experienced it. Both figures vary significantly according to socio-economic circumstances (as reflected by number of books in the home); less advantaged students report lower levels of participation in outdoor learning at school or wanting more opportunity to do so. Perhaps more concerning are the discrepancies among students who spend time in nature outside of school; of the 48% who reported having done so, most were socio-economically advantaged. More advantaged students were also more likely to report wanting to engage with the outdoors outside of school grounds (63% as compared with 56% of all students). This is also reflected in how students articulate their desire to protect the environment, with less advantaged students being less likely to understand ways of, or express a desire to, protect the environment. This is perhaps not surprising when previous research has found a link between nature connection and pro-environmental behaviour (e.g., [72,73]).

To shift the education system towards promoting human-to-nature connectedness and reducing inequalities, as well as more directly responding to the suggestions articulated by students in free-text comments, we suggest the need to embed opportunities for outdoor learning and nature engagement for children and young people of all ages within and beyond the formal curriculum. This research builds on prior evidence that outdoor learning as facilitated by the school contributes to nature connection (e.g., [74]), and that engaging with nature in this way correlates with participation and action for protecting the environment (e.g., [75]). And as this study and others demonstrate, students from lower socio-economic circumstances are significantly less likely to convey a sense of nature connection (e.g., [76]). Bearing in mind this distinction and that 85% of the UK population currently reside in urban areas with minimal access to nature (World Bank Group [77]), school provision for fostering nature connection is, therefore, a crucial component to delivering equitable and engaging CCSE. Our data emphasises outdoor learning as a means of achieving nature connection for all students, which would contribute to individuals’ pro-environmental behaviour and greater health and wellbeing. This should span the curriculum, and include activities, such as field trips, activities inspired by forest school and outdoor adventure education, teaching traditional and non-traditional subjects in natural environments, or arts-in-nature practices [78,79].

## Conclusion

At its launch, the CAR stated a commitment to ‘a broader, richer, cutting-edge curriculum that drives high and rising school standards and sets all young people up for life and work will be central to the government’s vision for education’ ([46], n.p). In this way, it offers an opportunity for CCSE to be embedded across the curriculum, including opportunities for children and young people to engage with and through nature and the environment outdoors. Students in our survey experienced CCSE differently but, when asked how they would improve it, articulated a clear desire for CCSE which afforded more opportunities for exploring climate change and sustainability across a wider range of subjects; opportunities for school-based and broader community action; and physical engagement with nature and the outdoors. However, successful integration of CCSE into formal education cannot be achieved by curriculum reform alone. It is fundamentally dependent on the capacity, confidence and ongoing professional development of teachers. Teachers are not only facilitators of an improved curriculum; they are important agents of change who shape how it is implemented in classrooms [80]. Currently, the ways in which CCSE is incorporated into the school curriculum vary considerably and depend largely on the expertise and priorities of individual teachers (and their schools); without adequate training and guidance, this leads to vast inconsistencies in delivery across the country [14]. This study shows that such inequalities result in unequal experiences for students, with those from disadvantaged socio-economic backgrounds being less likely than their more advantaged peers to enjoy learning about, or see the value of, CCSE. They were also less likely to be encouraged to spend more time outdoors by their parents and less likely to engage with activities beyond formal classroom learning. These findings accord with existing research that has shown patterned provision of extra-curricular activities within education, including where young people with fewer resources face barriers around participation [66]. Therefore, we argue that any serious commitment to embedding CCSE into the National Curriculum must be matched with a robust strategy for teacher learning and support. This includes discipline-specific training that equips educators with the tools and critical understanding necessary to deliver meaningful CCSE in their respective subject areas. Without this investment in teachers, CCSE risks remaining inconsistent and inequitable, thus failing to meet aspirations set out by policy or the needs expressed by young people themselves.

## Data Availability

The datasets generated during and/or analysed during the current study are available from the corresponding author on reasonable request.

## Note

1 See Maisuria [10] for details pertaining to each of the UK’s four countries (England, Scotland, Wales and Northern Ireland) and their respective educational jurisdictions.
